# Effects of honeybush (*Cyclopia subternata*) extract on physico‐chemical, oxidative and sensory traits of typical Italian salami

**DOI:** 10.1002/fsn3.1509

**Published:** 2020-03-24

**Authors:** Paula Smit, Marco Cullere, Antonella Dalle Zotte, Stefania Balzan, Louwrens Christiaan Hoffman, Enrico Novelli

**Affiliations:** ^1^ Department of Animal Medicine, Production and Health Padova University Agripolis Legnaro (PD) Italy; ^2^ Department of Food Science University of Stellenbosch Stellenbosch South Africa; ^3^ Department of Comparative Biomedicine and Food Science Padova University Agripolis Legnaro (PD) Italy; ^4^ Department of Animal Sciences University of Stellenbosch Stellenbosch South Africa; ^5^ Centre for Nutrition and Food Sciences Queensland Alliance for Agriculture and Food Innovation (QAAFI) Health and Food Sciences Precinct University of Queensland Coopers Plains Qld Australia

**Keywords:** *Cyclopia subternata*, honeybush extract, Italian type salami, lipid oxidation, natural antioxidant, ripening, sensory traits

## Abstract

Honeybush (*Cyclopia subternata* Vogel) is an indigenous South African shrub enjoyed as hot brewed tea. “Unfermented” honeybush is also a potential antioxidant bioactive extract for foodstuffs due to its polyphenol content. The effect of “unfermented” honeybush extract (Hob; 0.5%) was evaluated in typical Italian salami and compared with nitrate (Nit; 100 mg/kg) and a control (Ctl; without nitrate or honeybush). After 35 days of ripening, Hob had a higher (*p* < .01) water activity (0.928), compared with Ctl (0.923) and Nit (0.924). Final pH (5.35–5.24) was not affected by treatments. Lower lipid oxidation was observed in Hob and Nit treatments (*p* < .001) compared with Ctl. Internal color and odor intensity were similar among treatments. Salami with honeybush extract had less spontaneous outer surface mold growth whereas the Ctl showed intermediate growth (*p* < .05). Honeybush extract seems a promising natural ingredient with antioxidant action.

## Graphical abstract

Honeybush (*Cyclopia subternata* Vogel) is an indigenous South African shrub enjoyed as hot brewed tea. The effect of unfermented honeybush extract (Hob; 0.5%) was evaluated in typical Italian salami and compared with nitrate (Nit; 100 mg/kg) and control (Ctl; without nitrate or honeybush). After 35 days of ripening, Hob had a higher (*p* < .01) water activity (0.928), compared with Ctl (0.923) and Nit (0.924). Final pH (5.35–5.24) was not affected by treatments. Lower lipid oxidation was observed in Hob and Nit treatments (*p* < .001) compared with Ctl. Internal color and odor intensity were similar among treatments.

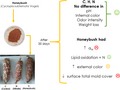

## 1 INTRODUCTION

Typical Italian salami is characterised as a long ripened, dry‐cured, and fermented sausage, usually covered in surface molds and generally not smoked. These Mediterranean type sausages are known and appreciated for their mildly acidic taste, seldom reaching a final pH lower than 5 (Holck, Axelsson, McLeod, Rode, & Heir, 2017). Gray‐white outer surface mold growth is a desirable phenomenon in these salami, contributing to product aesthetics and importantly, a particular sensory profile achieved through complex extracellular protease and lipase actions (Flores, 1997). Mold colonization can either occur spontaneously from the equipment and environment, or by deliberate dipping or spraying (Flores, 1997). Furthermore, surface mold growth mostly consisting of *Penicillium nalgiovense* (Lücke, 2000) contributes to a number of advantages, such as protection against oxygen exposure, inhibiting the growth of undesired and potentially dangerous molds, and modulating the weight loss process (Lücke, 2000).

Traditional, ancient processing methods based on family knowledge paved the way for standardising industrial salami production, establishing the use of functional starter culture strains, the direct addition of nitrate and/or nitrite preserving salts and the use of controlled environmental ripening chambers (Leroy, Geyzen, Janssens, Vuyst, & Scholliers, 2013). Today, salami are valued for their organoleptic traits and their robustness for use in different culinary scenarios in many countries. Despite their popular status, modern‐day consumer demand for cleaner labelled and less processed foodstuffs is the driving force for new product development and processing innovation, not limited to, but including traditional meat products (Weiss, Gibis, Schuh, & Salminen, 2010). Regarding dry‐cured meats, an abundance of research investigated the possible adverse health effects of the curing agents nitrate and more so nitrite, often used in salami production. These additives are involved in the synthesis of N‐nitrosamines (NAs), some of which are genotoxic and classified as probable human carcinogens (De Mey, Maere, Paelinck, & Fraeye, 2017; Herrmann, Duedahl‐Olesen, Christensen, Olesen, & Granby, 2015; Herrmann, Duedahl‐Olesen, & Granby, 2015; IARC, 1978). Coagulase‐negative *Staphylococci* (CNS) strains are commonly added to nitrate‐containing salami meat batters for their nitrate reductase action (Gøtterup et al., 2008; Hammes, 2012). Within this group, *S. xylosus* is frequently reported as the dominating strain isolated from spontaneously fermented salami (Cocolin, Manzano, Aggio, Cantoni, & Comi, 2001; Coppola, Iorizzo, Saotta, Sorrentino, & Grazia, 1997) and is commonly added as part of Mediterranean salami starter cultures (Leroy, Verluyten, & Vuyst, 2006).

Nitrate salts, commonly used in nonheat‐treated meat products, are rapidly reduced into nitrite during the first days of ripening. Nitrite is a multifunctional highly effective antimicrobial and antioxidant agent as well as an excellent compound providing a bright red color for which there is yet to be a single replacement (Honikel, 2008; Sebranek & Bacus, 2007). Although the scientific evidence associating nitrite with adverse human health effects is equivocal (Bedale, Sindelar, & Milkowski, 2016; Hord, Tang, & Bryan, 2009; Song, Wu, & Guan, 2015), researchers are attempting to omit or partially replace nitrate and nitrite in meat products by using novel, naturally derived compounds with inherent antimicrobial and/or antioxidant effects (Alirezalu et al., 2019; Gassara, Kouassi, Brar, & Belkacemi, 2016).

Honeybush (*Cyclopia subternata* Vogel, Family: *Fabaceae*, Tribe: *Podalrieae*) is an indigenous South African shrub enjoyed as hot brewed tea after “fermenting” the stems and leaves (De Beer et al., 2012; Joubert, Gelderblom, Louw, & Beer, 2008). *Cyclopia subternata* has proven to be rich in compounds with biological activity referable to a wide range of phenolic and nonphenolic compounds as well as glycosides especially in the “unfermented” state compared to the high temperature exposed “fermented” state. Among them, there are some flavanones (hesperidin, narirutin, eriocitrin, eriodictyol, and hesperetin the aglycone of hesperidin), flavones (luteolin and scolymoside), epigallocatechin gallate, and xanthones (mangiferin and isomangiferin). Even if the most abundantly present are mangiferin and hesperidin, it must be emphasize that the polyphenolic composition will vary according the harvesting season and processing methods to prepare the extracts. Although mangiferin (glycoside) seems to be one of the most effective compounds in quenching ABTS^•+^, the aglycones flavonoid are those more prone to inhibit lipid peroxidation (such as luteolin, hesperitin, and eriodictyol). The higher hydrophilicity due to the presence of the sugar residue can exclude mangiferin from the hydrophobic core of the membrane, where part of the lipid peroxidation take place (Joubert, Joubert, Bester, Beer, & Lange, 2011; Joubert, Richards, et al., 2008; Kamara, Brand, Brandt, & Joubert, 2004; Kokotkiewicz et al., 2012). However, Honeybush exhibits antioxidant capacity in both the “unfermented” state and the “fermented” state (Joubert, Gelderblom, et al., 2008). The potential of “unfermented” honeybush extract received attention as a possible bioactive food ingredient in 2000 and the processing of vacuum dried, “unfermented” honeybush tea was subsequently patented (De Beer & Joubert, 2002). Nowadays, “unfermented” honeybush is not only recognized as a beverage, but also as a potential extract for functional foods. 

Nonetheless, there is a lack of research on the potential of using honeybush in processed meats as a natural antioxidant. Based on these premises, the objective of the present experiment was to study the possible application of “unfermented” *C. subternata* extract in the manufacturing of Italian type salami and to compare it to a traditional recipe where nitrate was included. Physico‐chemical traits and sensory characteristics of the ready‐to‐eat product were evaluated.

## 2 MATERIALS AND METHODS

### 2.1 Honeybush extract

An “Unfermented,” vacuum dried *C. subternata* extract was supplied by the Agricultural Research Council (ARC),‐Infruitec‐Nietvoorbij, Stellenbosch University, South Africa and prepared as previously described (Schulze et al., 2016). Briefly, plant material was subjected to preheated purified water (90°C, 30 min in a 1:10 m/v ratio) in a percolator‐type extraction vessel. The extract was subsequently drained, centrifuged, concentrated, vacuum dried (40°C, 24 hr), and frozen (−20°C) in moisture, light, and oxygen impermeable packaging. The final extract (in the form of a fine, tanned colored powder) had a pH of ~5.0 and did not exhibit buffering capacity.

### 2.2 Salami production

Salami manufacturing was carried out at the meat laboratory of Veneto Agricoltura (Thiene, Vicenza, Italy). Fresh pork (shoulder and belly) was purchased from a local butcher the same day of the salami manufacturing.

The lean meat and fat (approximatively 70:30 w/w) were minced through a disk with 6 mm holes (mincer model TCS 32, Roberto Cavalli Meat Processing Machinery Srl) and mixed using an electrical kneading machine (model IMP 50, Roberto Cavalli Meat Processing Machinery Srl). A total of 33.5 kg of meat batter were divided into three subsamples. Common ingredients were added to each subsample, including salt (2.5%), dextrose (0.5%), and starter culture mixture (0.2%). The starter culture was in a freeze‐dried powder form and contained a total amount of 305 × 10^9^ ± 0.5 log colony forming units (CFU) in approximately equal amounts of *Staphylococcus xylosus*, *Lactobacillus sakei,* and *Pediococcus pentosaceus* (SA13‐100M; BIOAGRO Srl). The three respective subsamples were mixed until homogenous and weighed 11.36 kg each afterward. Each subsample was divided into three treatments of 3.8 kg each: Ctl (mixture without addition of other ingredients); Nit (Ctl plus 100 mg/kg of potassium nitrate and 0.05% of ascorbic acid); and Hob (Ctl plus 0.5% honeybush extract, w/w).

Each batch was manually mixed and stuffed using a vertical bagging machine (model ID 25V, Roberto Cavalli Meat Processing Machinery Srl) into bovine casings (60–65 mm in diameter, 9 m in length). A total of 68 salami (*n* = 23 Ctl, *n* = 23 Nit, *n* = 22 Hob) were obtained, with an average fresh weight of 530 g each.

The salami were placed in a controlled ripening chamber (Majolo^®^ Plus 100 Seasoning Controller). The drying step took 6 days, at a temperature decreasing from 21°C ± 1°C (first day) to 16°C ± 1°C (last day); the relative humidity (RH) varied between 65% and 80%. Afterward, the ripening step took 29 days with the temperature set at 14°C ± 1°C and RH set at 80% ± 10%. A final weight loss of 35% was used as the indicative end‐point of the ripening process (value that was reached after 35 days of processing).

### 2.3 Physico‐chemical parameters

#### 2.3.1 Weight loss, water activity (a_w_), and pH

Weight loss (%) was evaluated on days 5, 14, 21, 32, and 35. The *a_w_* was measured on the end product using an Aqua Lab 4 TEV (METER Group, Inc.)*.* The final pH was measured (Knick Portamess 911) by inserting a glass probe (Xerolyt Plus electrode, Mettler Toledo) in the salami center and underneath the casing.

#### 2.3.2 Moisture content and oxidative status

The moisture content (%) of the end product was determined gravimetrically (AOAC, 1990). The oxidative status was measured on the end product (35 days) using the TBARs (Thiobarbituric Acid Reactive substances) method as described by Botsoglou et al. (1994). To highlight the potential antioxidant action of honeybush extract, at the end of processing all the salami were sliced (*n* = 10 slices of 2 mm thickness), packaged on a rigid tray covered with a PVC pellicule (KOEX 412), and stored in a display cabinet under alternating exposure to fluorescent light (12 hr dark and 12 hr light; Osram Natura De Luxe L36 W/76e1) at 4°C ± 1°C for 4 days. All the slices of each tray were ground together using a knife mill (Grindomix GM200, Retsch) from which two aliquots were taken for analysis and the results expressed as malondialdehyde (MDA) equivalents (mg MDA/kg product).

### 2.4 Sensory analysis

Salami were submitted to a descriptive sensory analysis, to detect possible differences among the experimental treatments (Ctl versus Nit versus Hob). Due to lack of microbial testing of the end product, the samples were only orthonasally.

The sensory analysis was performed by a eight member expert panel (Istituto per la Qualità e le Tecnologie Agroalimentari, Laboratorio Analisi Sensoriale – Veneto Agricoltura), qualified according to ISO 8586:2012 and that had experience with sensory analysis (ISO 13299:2016) on various food matrices.

All judges who perform tests with accredited methods undergo training every 3 years. Some of the sensory tests were conducted on the whole sample: (a) intensity of the red color of lean meat visible through the casing, (b) the percentage of casing coverage by the mold, and (c) the recognizable color of the mold. After slicing the salami, the following evaluations were conducted: (d) the red color intensity of the inner lean meat and (e) the odor intensity. The tests (a), (b), (c), and (d) were conducted under a white light, whereas for (e) test a red light was used. The tests (a), (d), and (e) were conducted according to a continuous scale of increasing intensity (1 = the lowest score for each attribute and 9 = the highest score for each attribute). Conversely, the tests (b) and (c) were performed using a four‐category classification: 1 = 0%, 2 = 0%, 3 = 60%, and 4 = 100% for mold cover (Figure S1a), and white, gray, yellow, and green for mold color, respectively. If the case, panellists could mark more than one color. To implement the ability of the judges with the test (a), a preliminary training with three reference samples was carried out (Figure S1b). All the tests were done in a laboratory where the temperature was set at 20°C (ISO 8689:2007). A total of 15 salamis/treatment were used, and 3 days of analysis were scheduled (5 salamis/treatment/session). Samples were identified by a random three‐digit code, different for each sample and for each panellist. Data acquisition and processing were performed using FIZZ software (Biosystèmes France) installed in eight terminals in the tasting booths of the laboratory. After an initial assessment test of the repeatability of the judges' evaluations, two of the eight panellists were excluded from the elaboration of the results.

### 2.5 Statistical analysis

Data were analyzed using the GLM (general linear model) procedures of SAS 9.3 statistical software package for Windows (Cary, NC, U.S.). Salami weight loss and physico‐chemical traits were analyzed by day of analysis through a one‐way ANOVA that considered the treatment as fixed effect and batch as a block effect. A two‐way ANOVA was applied to detect any treatment effect on the salami sensory scores, considering the treatment, the assessors, and their interaction as fixed effects. Least square means were obtained, and post hoc pairwise comparisons were performed using the Bonferroni correction. Significance was considered at 5% confidence level. Surface mold cover percentage was analyzed with a one‐way nonparametric ANOVA (Kruskal–Wallis test), and the mold color evaluation frequencies were evaluated through a chi‐square test using the Marascuilo procedure.

## 3 RESULTS AND DISCUSSION

### 3.1 Weight loss, moisture content, a_w_, pH, and TBARS

All the salami reached the final desired weight loss (35%) within 35 ripening days with no significant difference among treatments over time as shown in Table 1. This weight loss range is often used as a quality check indicator for a commercially ready‐to‐eat Italian type salami (Cullere, Dalle Zotte, & Hoffman, 2012; Feiner, 2016). However, the salami *a_w_* at the end of the ripening was in the range 0.923–0.928, slightly higher than 0.92 which is considered as the safety threshold to inhibit the growth of *Listeria monocytogenes* (Roccato et al., 2017). However, product safety is not solely dependent on this one microbial hurdle; in fact, a combination of a pH of 5.2 and *a_w_* below 0.95 can also ensure microbial safety (UCFM, 2017), which is similar to the conditions that characterised the salami in the present trial. The honeybush‐treated salami (Hob) had a higher (*p* < .01) overall *a_w_*, compared with the Ctl and the Nit treatments (Table 2). A similar observation was noted when a freeze‐dried rooibos extract was used in the manufacturing of ostrich salami (Cullere, Hoffman, & Dalle Zotte, 2013). This finding was also partially supported by the moisture content resulting in the samples: the values observed for the Hob group were significantly higher (*p* < .01) compared with those of the Ctl, but similar to those of the Nit‐treated salami (Table 2). Therefore, further investigations are needed to check whether in particular Hob extract may have a direct or indirect effect on water retention into a fermented meat product. Independently to the experimental treatment, the moisture content of the salami in the present study (Table 2) was within the range of 24.3%–53% moisture, reported for typical commercial Italian salami (Zanardi, Ghidini, Conter, & Ianieri, 2010). Salami water loss, *a_w_*, and moisture content highlight the successful evolution of the drying‐ripening process.

**Table 1:** Effect of “unfermented” honeybush (*Cyclopia subternata*) extract compared with nitrate inclusion in Italian type salami: initial weight of salami and weight loss after 5, 14, 21, 32, and 35 days of processing

	Ctl	Nit	Hob	RSD^1^	Significance
	(*n* = 23)	(*n* = 23)	(*n* = 22)		
Initial weight (g)	521	518	541	67.1	ns
Weight loss (%)					
Day 5	13.3	13.5	13.4	1.39	ns
Day 14	23.6	23.7	23.3	1.66	ns
Day 21	29.0	28.8	28.4	1.77	ns
Day 32	34.5	34.4	33.8	1.88	ns
Day 35	35.7	35.5	35.0	1.92	ns

Note

ns = *p *> .05; Ctl = control (without the addition of nitrate or honeybush); Nit = nitrate (100 mg/kg); and Hob = honeybush (0.5% w/w).

^1^ Residual standard deviation.

**Table 2:** Effect of “unfermented” honeybush (*Cyclopia subternata*) extract compared with nitrate inclusion in Italian type salami: pH, moisture content, activity water (*a_w_*), and TBARs expressed as MDA quantity measured in the end product

	Ctl	Nit	Hob	RSD^1^	Significance
	(*n* = 23)	(*n* = 23)	(*n* = 22)		
pH‐final (inner)	5.35	5.28	5.24	0.13	ns
pH‐final (outer)	5.63	5.55	5.54	0.13	ns
Moisture (%)	33.5^a^	34.7^ab^	35.3^b^	0.37	**
*a_w_*	0.923^a^	0.925^a^	0.928^b^	0.004	**
MDA^2^ (mg/kg meat)	1.34^a^	0.41^b^	0.48^b^	0.09	***

Note

ns = *p *> .05; ** = significant (*p* < .01); *** = highly significant (*p* < .001).

^a‐b^Different superscripts in the same row indicate a significant difference.

Ctl = control (without the addition of nitrate or honeybush); Nit = nitrate (100 mg/kg); and Hob = honeybush (0.5% w/w).

^1^ Residual standard deviation.

^2^ Malondialdehyde.

In general, the pH of fresh salami batter drops during fermentation to an extent depending on the set environmental parameters, but also on the inoculated starter cultures, mainly due to the conversion of carbohydrates to lactic acid by lactic acid bacteria (Greco, Mazzette, Santis, Corona, & Cosseddu, 2005). In the present trial, the initial average pH of the raw meat batters were 5.50 (Ctl), 5.46 (Nit) and 5.51 (Hob), and after 35 days of processing, the final pH measured in the salami center was 5.24–5.35 with no significant differences ascribable among the treatments (Table 2). The pH range detected in the final product is typical of mildly acidic Mediterranean salami (Holck et al., 2017) and coherent with values indicated for commercial Italian salami (5.15 ≤ pH ≤ 6.83) (Zanardi et al., 2010).

Although some polyphenolic compounds present in honeybush, such as hesperidin and its active metabolite hesperetin, mangiferin, and luteolin exhibited antimicrobial properties against Gram‐positive bacteria (Biswas, Sen, Roy, Maji, & Maji, 2015; Dong et al., 2017; Ganeshpurkar & Saluja, 2019; Iranshahi, Rezaee, Parhiz, Roohbakhsh, & Soltani, 2015; Mazlan et al., 2019; Singh, Tiwari, Sinha, Danta, & Prasad, 2012), the addition of 0.5% honeybush extract did not influence the final pH negatively. Therefore, it is hypothesized that the extract had no effect on the lactic acid bacteria growth (not analyzed), which is primarily responsible for the pH drop in this type of meat product, although this should be evaluated in future studies. Results of the experiment showed that the center of the salami was slightly more acidic than underneath the casing, for all treatments. This was expected due to lactic acid metabolization and subsequent ammonia production by the molds located on the outer salami surface (Flores, 1997). The pH gradient becomes shallower or disappears as the ripening process' duration increases. The samples of the present study were subjected to a 35‐day of drying and ripening, which is not a long period for salami of 530 g weight.

The addition of 0.5% honeybush extract exerted the same protective effect of nitrate regarding lipid oxidation. In fact, both Hob and Nit treatments significantly (*p* < .001) lowered the salami MDA content (mg MDA/kg product) compared with the Ctl group (Table 2), thus confirming the antioxidant effect of honeybush previously reported in literature (Joubert, Gelderblom, et al., 2008). It is also worth highlighting that both the nitrate (0.41 mg MDA/kg) and the honeybush treatments (0.48 mg MDA/kg) resulted in lower TBARS than the recommended sensorial detected rancidity threshold of 1–2 mg MDA/kg (Watts, 1962 as cited by Love & Pearson, 1974). Both Nit and Hob resulted in much lower TBARS values compared with 0.01% added essential coriander oil (0.64 mg MDA/kg) and 0.01% synthetic antioxidant butylated hydroxytoluene BHT (1.06 mg MDA/kg), measured in typical Italian salami after 35 days of storage (Marangoni & De Moura, 2011). Similar to what was noted in the present experiment, other natural antioxidants proved to be highly effective in lowering the degree of oxidation in dry‐cured sausages, providing even better outcomes than the widely used synthetic antioxidant, BHT (Lorenzo, González‐Rodríguez, Sánchez, Amado, & Franco, 2013). As an example, “fermented” rooibos extract in ostrich salami caused a reduction in the oxidative status compared with the control group without any rooibos addition (Cullere et al., 2013).

### 3.2 Sensory analysis

The present experiment is the first to assess the impact of a honeybush extract on salami sensory characteristics. Previous research characterising the sensory profile of *C. subternata* summarized it as “fynbos‐floral” (fine‐leaved plants endemic to the Western Cape and Eastern Cape, provinces of South Africa) and “fruity‐sweet” lacking in bitter taste as opposed to other potential bitter counterparts like *C. genistoides* and *C. longifolia,* especially in the “unfermented” form (Alexander, Beer, Muller, Rijst, & Joubert, 2019; Erasmus, Theron, Muller, Rijst, & Joubert, 2017). For this reason, it was concluded that this specific *Cyclopia* species could be successfully applied in the manufacturing of food products. Even if the present experiment did not consider salami flavor and taste, results showed that internal color and odor intensity were similar in the Ctl, Hob, and Nit groups (*p* > .05; Table 3). The fact that no differences were found in the internal salami color indicated that the honeybush was neither more effective than the control, nor less effective than the nitrate in creating a desirable, attractive internal salami color. The only exception was regarding the external salami color, for which a tendency was observed toward a higher intensity for the Nit group (*p* < .1). The similar internal color of salami might be due to the role of the added coagulase‐negative staphylococcus (*S. xylosus*) as part of the starter culture mixture: extracellular bacterial nitric oxide synthase (NOS) produced by *S. xylosus* could have converted metmyoglobin into the cherry‐red nitrosomyoglobin in the absence of added nitrate or nitrite (Li, Kong, Chen, Zheng, & Liu, 2013; Ras et al., 2018). This could explain why even the Ctl and the Hob salami without nitrate were similar in internal color intensity, as determined by the sensory panel (Table 3). In past research considering a similar product, the incorporation of freeze‐dried and pulverized “fermented” rooibos extract into different processed meat products from ostrich (Hoffman, Jones, Muller, Joubert, & Sadie, 2014), ungulates (Jones, Hoffman, & Muller, 2015), and rabbit (Cullere et al., 2019) meat species highlighted an effect on the sensory attributes of the respective products. Depending on the type of product, the effects were positive (mitigation of the game meat flavor), neutral (rabbit meat with 0.5% inclusion level), or negative (rabbit meat with 1% and 2% inclusion levels); in all cases, a dose‐dependent effect was observed. For these reasons, further research evaluating the impact of the honeybush extract on the complete sensory profile of the treated meat product is of utmost importance for a possible practical application of this natural ingredient.

**Table 3:** Effect of “unfermented” honeybush (*Cyclopia subternata*) extract compared with nitrate inclusion in Italian type salami on the sensory traits: external color, internal color, and odor intensity evaluated on the end product

	Ctl	Nit	Hob	RSD^1^	Significance
	(*n* = 15)	(*n* = 15)	(*n* = 15)		
External color	5.34	5.80	5.42	1.35	†
Internal color	4.93	4.92	4.88	1.05	ns
Odor intensity	6.09	6.16	6.13	0.59	ns

Note

† = *p* < .1; ns = *p *> .05; for each attribute a continuous scale ranging from 1 (low intensity) to 9 (high intensity) was used.

Ctl = control (without the addition of nitrate or honeybush); Nit = nitrate (100 mg/kg); and Hob = honeybush (0.5% w/w).

^1^ Residual standard deviation.

**Figure 1:**
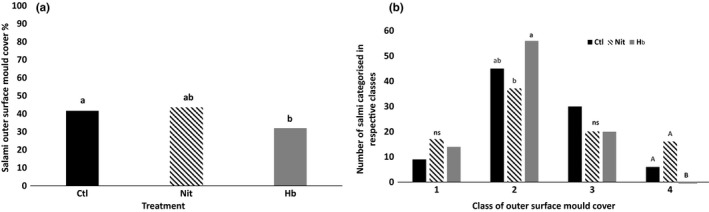
(a) Effect of “unfermented” honeybush extract (*Cyclopia subternata*) compared to nitrate inclusion on the spontaneous outer surface mold cover percentage of typical Italian salami (general mean percentage according to treatments); (b) effect of “unfermented” honeybush extract (*Cyclopia subternata*) compared to nitrate inclusion on the spontaneous outer surface mold cover percentage of typical Italian salami (data are showed according to the four classes of mold covering percentage: class 1 = 0%, class 2 = 30%, class 3 = 60%, and class 4 = 100% of coverage). Ctl = control (without the addition of nitrate or honeybush); Nit = nitrate (100 mg/kg ); and Hob = honeybush (0.5%w/w). (a) Different superscripts indicate a significant difference in outer mold cover % among treatments (*p* < .05); (b) different superscript indicate significant differences in outer mold cover % among salami treatments (a,b: *p* < .05; A, B: *p* < .01)

Both yeasts and molds colonize the surface of salami; yeasts have a key role in the fermentation process, and molds are fundamental to provide a desirable appearance, contribute to technological functions and sensory characteristics of the final product (Spotti, Berni, & Cacchioli, 2008). Salami in the present trial were not inoculated/sprayed for mold development; thus, growth on the external surface of the product was spontaneous. The surface of Hob salami had significantly fewer moulds than Nit salami (*p* < .05) but not significantly different from Ctl (*p* > .05) (Figure 1a). Interestingly, in all three groups the majority of salami fell in classes 2 (30%) and 3 (60%) surface mold cover, whereas classes 1 (0%) and 4 (100%) were poorly represented (Figure 1b). Into class 2 (30% covered), the Hob group had more salami compared to Nit (*p* < .05), but not significantly different from Ctl (*p* > .05). Conversely, considering class 4 (100% covered), the situation was reversed with Hob salami showing the lower value (0) compared with Nit and Ctl (*p* < .05). Considering characterisation of mould colour (Table 4), it could be deduced that white‐grey moulds proliferated better on the honeybush‐treated salami compared to the Ctl (*p* < .05), whereas the Nit group showed intermediate values. It was initially hypothesized that the lower mold cover percentage of Hob salami compared with Nit could be attributable to the demonstrated antimicrobial effect of hesperidin as well as that of mangiferin, respectively a flavanone and a xanthone contained in the honeybush extract (Iranshahi et al., 2015). However, the fact that Ctl and Hob salami did not differ in this sense suggested that other mechanisms, which would require an in‐depth investigation, may have played a role.

**Table 4:** Effect of “unfermented” honeybush (*Cyclopia subternata*) extract compared with nitrate inclusion in Italian type salami on mould color: percentage of samples on the total number of salami having at least 1 type of mould

	Ctl	Nit	Hob	*χ* ^2^	Significance
	(*n* = 15)	(*n* = 15)	(*n* = 15)		
White	23.9	13.8	20.6	2.1955	ns
White‐gray	17.9^b^	23.1^ab^	39.7^a^	8.5130	*
White‐yellow‐gray	14.9	15.4	6.35	3.0680	ns
White‐green‐gray	8.96	7.69	7.94	0.0788	ns

Note

*χ* ^2^ = chi‐square; ns = *p *> .05; * = *p* < .05.

Ctl = control (without the addition of nitrate or honeybush); Nit = nitrate (100 mg/kg); and Hob = honeybush (0.5% w/w).

^a,b^Different superscripts in the same row indicate a significant difference (*p* < .05).

## 4 CONCLUSIONS

Based on the results of the present research, the tested honeybush (*C. subternata*) extract seems promising as a natural ingredient intended for salami manufacturing. The extract was able to counteract the process of fat oxidation to a similar extent as the widely used nitrate but significantly lower than the Ctl batch and guaranteed satisfactory visual traits at a specific inclusion level. Further research should focus on the optimization of the salami drying‐ripening process to ensure optimal water activity of the final product. Furthermore, an in‐depth evaluation of the microbial composition of the end product (lactic acid bacteria and yeast) as well as to characterise the surface molds would be of utmost importance to ensure product quality and safety.

## CONFLICT OF INTEREST

The authors declare that they do not have any conflict of interest.

## ETHICAL STATEMENT

This article does not contain any studies with human participants or animals performed by any of the authors.

## Supporting information

Fig S1
