# Supplementary material for: Effects of honeybush (Cyclopia subternata) extract on physico‐chemical, oxidative and sensory traits of typical Italian salami
Source: Food Sci Nutr. 2020 Mar 24;8(5):2299–306. doi: 10.1002/fsn3.1509 (PMC7215206; doi:10.1002/fsn3.1509)
Supplement: Supplementary file 1 — Fig S1 [file FSN3-8-2299-s001.pdf]

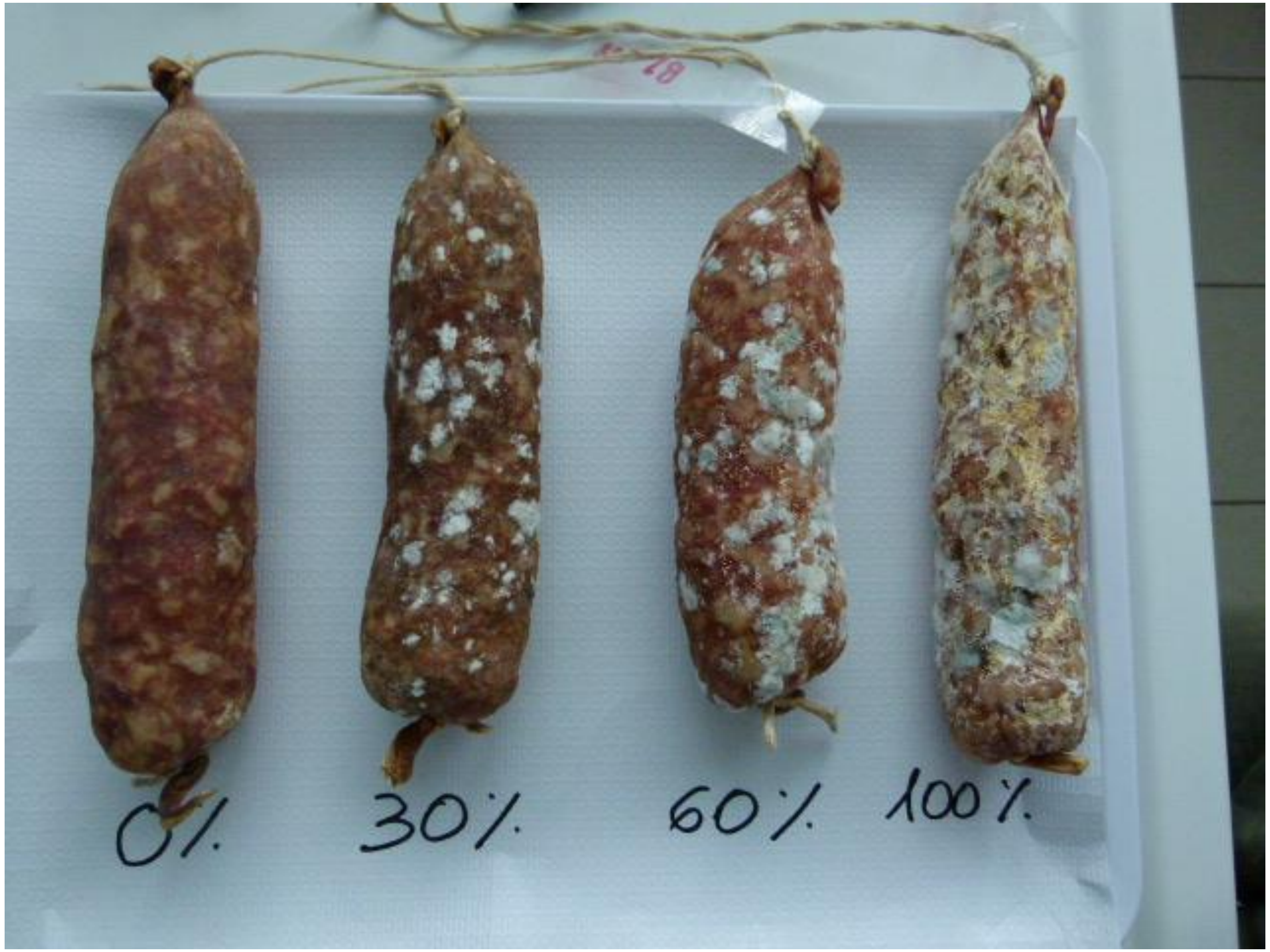

Figure A supp. inf. Samples of salami used for training in the evaluation of mould coverage according to four classes (0, 30, 60 and 100%)

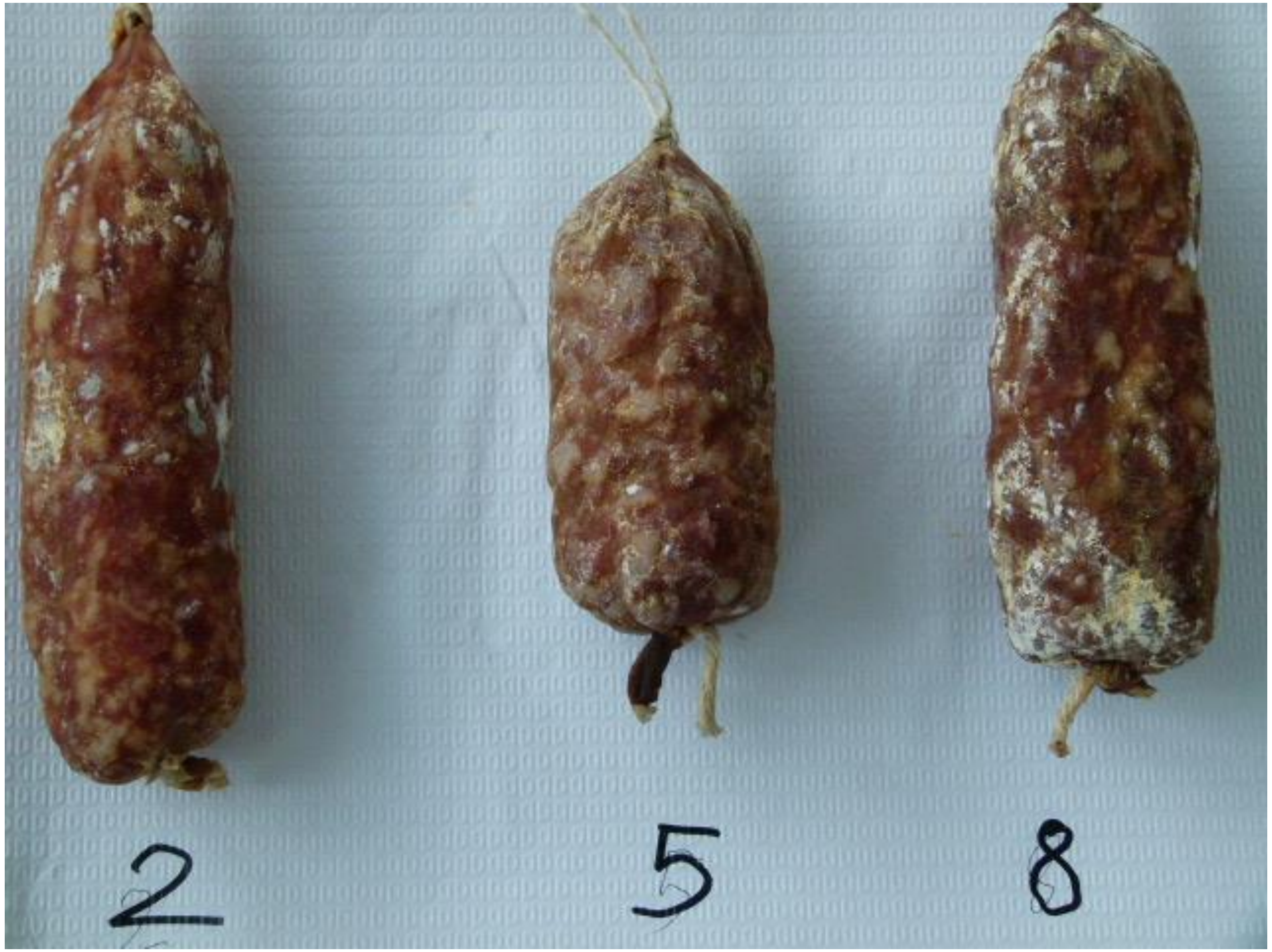

Figure B supp. inf. Samples of salami used for training in the evaluation of intensity of the red colour of lean meat visible trough the casing (continuous scale of increasing intensity, 1=low and 9=high)
